# Insights into Early Steps of Decanoic Acid Self-Assemblies under Prebiotic Temperatures Using Molecular Dynamics Simulations

**DOI:** 10.3390/membranes13050469

**Published:** 2023-04-28

**Authors:** Romina V. Sepulveda, Christopher Sbarbaro, Ma Cecilia Opazo, Yorley Duarte, Fernando González-Nilo, Daniel Aguayo

**Affiliations:** 1 Center for Bioinformatics and Integrative Biology, Facultad de Ciencias de la Vida, Universidad Andres Bello, Av. República 330, Santiago 8370146, Chile; 2 Instituto de Ciencias Naturales, Facultad de Medicina Veterinaria y Agronomía, Universidad de Las Américas, Manuel Montt 948, Providencia 7500000, Chile; 3 Agricultura Digital, Servicio Agrícola, Salinas y Fabres S.A., Ruta 5 Sur, Parcela 165, Hijuela Larga, Paine 9540000, Chile

**Keywords:** prebiotic membranes, decanoic acids, peptide–membrane interactions, molecular dynamics simulations

## Abstract

The origin of life possibly required processes in confined systems that facilitated simple chemical reactions and other more complex reactions impossible to achieve under the condition of infinite dilution. In this context, the self-assembly of micelles or vesicles derived from prebiotic amphiphilic molecules is a cornerstone in the chemical evolution pathway. A prime example of these building blocks is decanoic acid, a short-chain fatty acid capable of self-assembling under ambient conditions. This study explored a simplified system made of decanoic acids under temperatures ranging from 0 °C to 110 °C to replicate prebiotic conditions. The study revealed the first point of aggregation of decanoic acid into vesicles and examined the insertion of a prebiotic-like peptide in a primitive bilayer. The information gathered from this research provides critical insights into molecule interactions with primitive membranes, allowing us to understand the first nanometric compartments needed to trigger further reactions that were essential for the origin of life.

## 1. Introduction

Modern biological membranes comprise a complex mixture of phospholipids that self-assemble into bilayers that embed molecules and proteins. Decanoic acid (DA) are monocarboxylic acids with single 10-carbon hydrocarbon chains (C10), which, despite being short-chain fatty acids (SCFAs), are long enough to have amphiphile properties [1] and form vesicular membranes under defined pH conditions, ionic composition, and concentration. Due to their ability to self-assemble in aqueous solutions, their discovery in meteorite samples [2,3,4,5], and their synthesis in prebiotic conditions such as the Fischer–Tropsch process, DA are described as plausible primitive precursors of protocells at the beginning of the prebiological stages of the chemical evolution process, where complex molecules and supramolecular arrangements appeared and served as primers to produce life [6,7,8].

The self-assembly of DA molecules is influenced by the surrounding environment. DA molecules optimize their interactions by maximizing the hydrogen bond networks between their hydrophilic headgroups and the surface solvent [9]. As a result, the tails of the DA molecules pack together while the polar head groups are exposed, giving rise to various supramolecular assemblies such as micelles (at pH >6), vesicles (at pH between 4 and 6), and oil phases (at pH from 3 to 4) at 25 °C [10,11]. DA must meet certain minimum requirements, such as a critical membrane concentration (CMC) greater than 0.43 mM and a molar ratio of DA:NaCl lower than 3:1 [12]. To gain insights into the self-assembly processes of amphiphiles under prebiotic conditions, scientists often turn to modern terrestrial microclimates, such as hydrothermal vents, as analogs for the environments that existed on earth 3 billion years ago. These environments were characterized by ocean temperatures ranging from 70 °C to 100 °C [13,14,15].

The characterization of biological membranes is fundamental to understanding how molecules are embedded, diffuse, or permeate into the cell. Similarly, changes (an increase or decrease) in the supramolecular arrangements of protocells could have facilitated the embedding of early polymerized amino acids [16,17,18], which later could have had an essential role in modulating the transport of elements to the internal compartment [19].

DA, like other SCFAs, can generate vesicular bilayer structures with geometries that depend on the balance of attractive and repulsive interactions between monomers and the environment. The supramolecular arrangements adopted by DA are also affected by temperature, which can modify their structure, permeability, fluidity, and how molecules interact inside them. Although prebiotic environmental conditions remain unclear, temperatures between 26 °C and 35 °C [19,20] are commonly expected to occur, which are near the phase transition temperature required to change from an ordered gel phase, where the hydrocarbon chains are fully extended and closely packed, to the disordered liquid crystalline phase, where the hydrocarbon chains are randomly oriented and fluid.

Today, molecular dynamics (MD) simulations allow us to understand the behavior of relatively large chemical systems, such as vesicles, at the atomic level. These simulations use energetic potentials and Newtonian equations of motion to describe molecular conformations and intermolecular forces (dispersion and electrostatic forces) geometrically and dynamically. A 10 nm DA vesicle in water has approximately 2000 atoms, of which an MD simulation can simulate hundreds of nanoseconds in a few days, which would be enough to study the rapid self-assembly kinetics and phase properties. Thus, MD simulations of DA assemblies are an alternative to studying the properties of primitive membrane models and give insights into the complex interactions between molecules and their environment, helping to shed light on how life appeared on earth [18,21,22,23,24,25].

This study aimed to gain insights into how temperature impacts the DA self-assembly processes and structure. Firstly, we used dynamic light scattering assays to characterize the size of DA vesicles under standard experimental conditions. Subsequently, we used molecular dynamics simulations to study a simplified homogeneous DA system, enabling further observation into the first steps of DA vesicle formation, stability, and permeability at temperatures ranging from 0 °C to 110 °C. Furthermore, we present several full-atom MD simulations of DA vesicle systems interacting with a prototype of a prebiotic-like peptide under different temperatures. These enabled us to explore how the thermotropic behavior of DA vesicles as a protocell model influences peptide insertion and helps us further understand life’s origins.

## 2. Methods

### 2.1. Preparation of Decanoic Acid Vesicles

Decanoic acid at 98% purity was purchased from Sigma-Aldrich (St. Louis, MO, USA). Vesicles of decanoic acid and decanoic acid/cholesterol were prepared by adding NaOH (1 M) dropwise to a solution of decanoic acid (0.1 M) at 30 °C, vortexing between additions, until the solution reached a pH of around 11. HCl (1 M) was added, and the pH was monitored until it reached the pKa of decanoic acid (pH = 4.9) [6]. At this point, the solution had a white turbidity. To determine whether the solvent medium has an impact on the formation of vesicles, suspensions were prepared in ultra-pure water and PBS buffer solution. To homogenize the size, the vesicle suspensions were bath-sonicated in a Biobase Digital ultrasonic cleaner (Model UC-08A) (Shandong, China) for 5 min at 30 °C.

### 2.2. Dynamic Light Scattering (DLS) and Transmission Electron Microscopy

The size, polydispersity, and zeta potential of sonicated decanoic acid vesicles were measured on Malvern Instruments (Malvern, Worcestershire, U.K.). Zetasizer nS was used with a measurement angle of 173° (standard for non-invasive back scatter), using PBS as the primary dispersant. The measurements were carried out in a chamber that was kept at a constant 25 °C temperature. Three measurements of each sample were taken, with the average result being noted.

The vesicle suspensions were examined using an electron transmission microscope, the Talos f200c G2 (Thermo Fisher Scientific, Waltham, MA, USA), to explore the morphology of the vesicle system. Uranyl acetate was used as a contrast agent, and a 10 μL aliquot of diluted vesicle sample was allowed to react with it.

### 2.3. Molecules Built and Systems Set Up

The protonated state of the decanoic acid molecule was built using the VMD molefacture module based on its pKa of 6.41 [10].

We built two initial molecular systems composed of 1000 decanoic acid units replicated in random places in a cubic box using the Packmol package [26]. The first set of molecular systems was as follows: system: DA, composed purely of decanoic acid molecules; and system: DAP, composed of decanoic acid molecules and a prebiotic-like peptide. A prebiotic-like peptide of 16 amino acids (SIAIAIAIAIAS) was built using VMD and located randomly in the molecular systems. The peptide composition was chosen based on the availability of hydrophobic amino acids in the prebiotic conditions [8].

Each molecular system was hydrated with TIP3P waters to a dimension of 12 × 12 × 12 nm^3^ in cartesian space, and we added salt to reach a concentration of 0.10 M NaCl, using VMD. The system comprises 190,000 atoms, where ~52,400 are water molecules. The decanoic acid concentration reached 1 M, which provides an affordable system able to reach intermolecular interactions in a sustainable computational time.

### 2.4. Molecular Dynamics Simulations

All MD simulations were performed using the Amber22 software (San Francisco, CA, USA) [27]. The systems were first energy-minimized for 5000 steps and equilibrated for 1 ns using an isobaric–isothermic ensemble (NPT) at 1 atm and 298 K (system DA-25), with the peptide backbone and DA headgroups restrained with 10 to 0.1 kcal/Mol Å^2^ to enhance homogenous solvation. Then, the system was simulated as a production simulation for a period of 320 ns without any geometrical restraints. To represent the effect of temperature on the DA bilayer phase, the DA-25 system was slowly cooled (25 °C to 0 °C) or heated (25 °C to 110 °C) using a temperature variation of 10 °C for a period of 40 ns prior to the production simulation, which lasted ~280 ns per temperature. Twelve starting configurations of peptide-containing systems were prepared and independently submitted to MD runs at the desired temperature. The temperature was maintained constant, employing Langevin dynamics with a damping coefficient of 1 ps^−1^. Periodic boundary conditions were applied to all simulations. The equations of motion were integrated with an effective time step of 2, 2, and 4 fs for bonded and short- and long-range nonbonded interactions, respectively. A 10 Å spherical cutoff for short-range nonbonded interactions was applied. The TIP3P model of water was used, and all simulations made use of the amberff19SB force field [28], and gaff2 was the base for the force field setting. The parameters and topology of decanoic acid were built using Amber22′s LEaP module [29].

### 2.5. Trajectory Analysis Tools

We calculated the radius of gyration from the headgroups (O1 and O2) of DA assemblies, which allowed us to infer the bilayer thickness and vesicle radius by subtracting the differences between the measurement from the vesicle center to the internal or external leaflet of the decanoic acid bilayer.

Next, we calculated the diffusion coefficient to understand the displacement of DA at different temperatures. The diffusion coefficients were computed from the Diffusion Coefficient Tool compatible with Visual Molecular Dynamics (VMD) and implemented by Giorgino et al. (2019) [30]. The calculation is based on the calculation of mean squared displacement (MSD), which defines M(τ) by a fixed time origin and lag time τ as the mean displacement over the interval:

M(τ) = ⟨|r(τ) − r(0)|^2^⟩ (1)

where r(τ) is the position of a particle at time τ, and the brackets are the average over all particles of the species (Equation (1)). Afterwards, the diffusion coefficient was measured through the Einstein relation:

D(τ) = M(τ)/2Eτ(2)

where E is the dimension of the system, which was calculated on axes x, y, and z (Equation (2)) [30]. The analysis interval used in the trajectories was 0.01 ns.

Considering the coefficient diffusion, we explored the bilayer water permeation under different temperature exposure through the solvent-accessible surface area (SASA).

In order to provide a fair analysis of the peptide insertion in the decanoic acid bilayer from DAP systems, the tilt angle between the peptide axis and the bilayer norm vector was calculated.

To improve the results, we incorporated the order parameter of decanoic acid molecules from every trajectory. The measurement was performed by reorientating each compound to the z axis to avoid the curvature for normal calculation [31,32].

S_CH_ = ⟨3 cos^2^ *θ* − 1⟩/2 (3)

Additionally, we analyzed the location and behavior of decanoic acid molecules along the trajectories through density maps that were calculated based on the headgroups of decanoic acids in two dimensions [33]. Finally, the radial distribution function in DAP systems resulted in the distribution of distances between the peptide and DA and aimed to complement the tilt angle results.

## 3. Results

### 3.1. Exploration of a DA Vesicle under Standard Conditions

**Figure 1:**
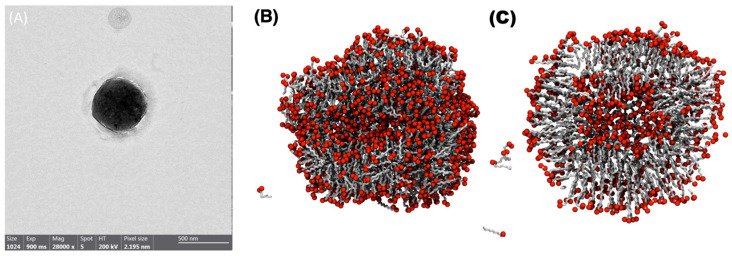
Experimental and theorical comparison of decanoic acid vesicles. (**A**) TEM image of decanoic acid vesicle prepared in ultra-pure water. (**B**,**C**) Snapshots after 300 ns of a decanoic acid molecular system at 25 °C.

DA’s capacity for self-assembly in aqueous solutions was determined by using dynamic light scattering and transmission electron microscopy. TEM revealed that DA forms mono- and multilamellar vesicles of different sizes under the experimental conditions assayed, in agreement with the size (Figure 1) and zeta potential (−13.8 ± 1.0 mV) experimental values. It is worth noting that a temperature of 298 K and pH of 6.4 were used to promote the vesicle formation, in agreement with experiments resembling the protocell environment [10]. Although the traditional method for creating vesicles and liposomes containing phospholipids requires organic solvents and a step of dehydrating and rehydrating a lipidic layer, our method enabled us to create the system directly in an aqueous solution without extrusion, with pH adjustment serving as the primary mechanism for vesicle formation [31], aiming to represent hydrothermal vent conditions. 

### 3.2. DA Spontaneous Aggregation into Vesicles

As described above, MD is based on solving the equations of motion of the different constituents of the system under consideration; thus, it is a valuable tool to characterize the formation mechanism, stability, structure, and interactions of molecular systems. In this study, MD was used to gain insights into the self-assembly process of DA molecules. The initial system comprises 1000 randomly placed DA monomers that rapidly form (10 ns) local clusters that coalesce into bilayers and transform into a spheroidal vesicular structure with encapsulated water. Figure 1B shows a snapshot of the resultant DA bilayer vesicle after a 300 ns MD simulation, which has a 7 nm diameter and a bilayer thickness of 1.5 nm at 25 °C. This result agrees with what was described using TEM and DLS and with the results of Namani and Walde (2005) [34]. The resulting rough vesicle formed in the simulation comprises 1000 lipids, 21% of them residing in the inner bilayer leaflet. Analysis of the DA-25 trajectory revealed a ripple phase at 25 °C, formed by gel-like and fluid-like coexisting DA patches, as evidenced by visual inspection and the motional behavior derived from order parameter results (Figure S1 in Supplementary Materials). According to the DA chain length, the lateral diffusion of monomers occurs rapidly compared with modern membrane lipids. Furthermore, although scarce, flip-flop events (6 in 300 ns) between leaflets were observed; the number of occurrences is higher than expected for phospholipid vesicles with similar simulation times [35]. These results support the formation of DA vesicles at 25 °C, thus generating a stable water-filled compartment. This condition is below the experimental melting temperature reported for DA, and the ripple phase observed at this temperature could be relevant to restrict small molecule permeation or the transmembrane diffusion required for the evolution of prebiotic systems.

### 3.3. Spontaneous DA Vesicle Formation in a Wide Range of Temperatures

Several theoretical studies suggest that thermal gradients present in hydrothermal vents (between 5 °C and 50 °C [36]) could support the accumulation and self-assembly of primitive amphiphiles. Accordingly, we used MD to gain insights into the phase behavior of DA vesicles as a function of temperature, aiming to characterize their structural changes and possible effects on the compartmentalization potential.

Table 1 summarizes the structural characteristics of the DA vesicles formed during the simulations at 0 °C to 100 °C, using the system at 25 °C as a starting configuration. Although the thickness of the membrane remains almost constant (from 1.4 nm to 1.5 nm), there are several temperature-dependent differences. According to DA Tm, at 40 °C the vesicle displays a spheroid shape with a defined water-filled inner compartment. According to the order parameter and DA monomer geometries, between 20 °C and 30 °C the bilayer displays a transitional state, with almost 80% of the inner-leaflet and ~30% of the outer-leaflet monomers adopting a fluid phase (Figure S1 in Supplementary Materials). As the temperature decreases, the vesicles gradually become larger, with both vesicle leaflets exhibiting a gel phase. Conversely, the simulated systems adopted a spherical geometry at temperatures above 50 °C, with a more diffuse and fluid DA membrane with both leaflets in the fluid phase (Table 1).

**Figure 2:**
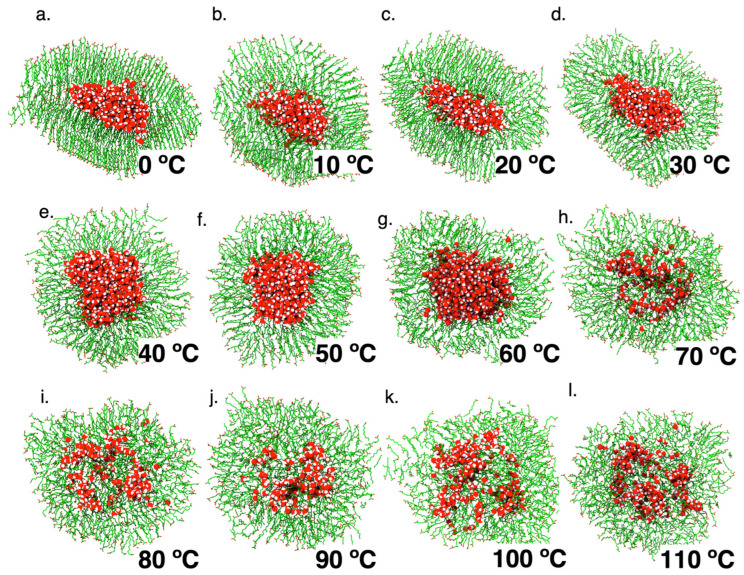
Description of molecular dynamics trajectories of decanoic acid systems under different temperatures. Decanoic acid molecules promote aggregation to form vesicles at different temperatures. Each picture exhibits a representative frame from the DA trajectory. Water molecules are depicted in red.

As mentioned above, an asymmetric behavior of the membrane leaflets occurs between 30 °C and 20 °C, below the transition temperature (Tm) described for DA vesicles. As shown in Figure 2, the curvature of the outer leaflet is smaller than the inner leaflet due to a monomer proportion imbalance and monomer packing (Figure S2 in Supplementary Materials). Accordingly, the rippled phase observed is linked to a favorable free energy state, with outer leaflet regions adopting a gel structure before the inner layer as the temperature decreases, increasing the van der Waals interactions and reducing the surface area accessible to the solvent at the headgroups exposed to the environment (Table 1, Figure S3 in Supplementary Materials).

Trajectory analyses also showed changes in the vesicle compartmentalization potential. Below the Tm, the water-filled compartment becomes more defined and oblong, without permeation to the environment of water molecules. On the other hand, as T > Tm, the increase in temperature results in less packing of the DA chains, which generates spaces that allow the permeation of water molecules. This leads to a smaller compartment volume, promoted by restructuring the inner leaflet of the bilayer. This observation agrees with new theories that relate the structure, fluidity, stability, and local curvature with the generation of the chemical potential required at the origins of life.

### 3.4. Peptide Interaction with Decanoic Acid Vesicles as a Key to Understanding the Encapsulation Process

**Figure 3:**
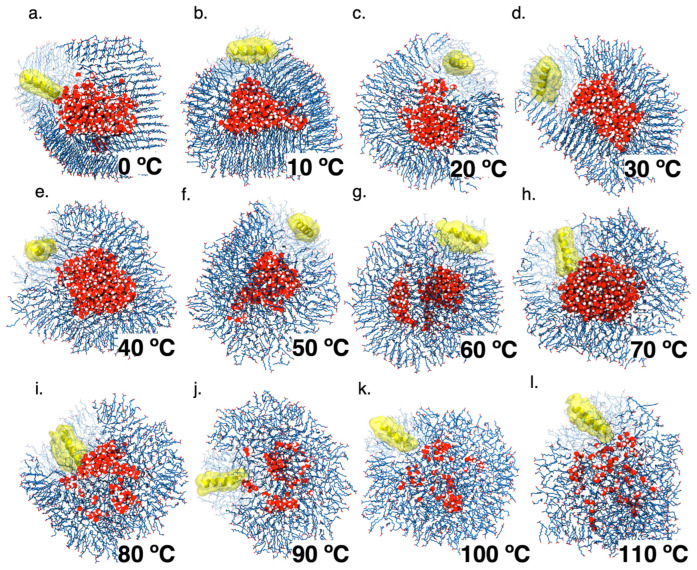
Description of molecular systems from decanoic acid molecules and peptide. The decanoic acid molecules are represented in blue, water as red spheres, and peptide prototype in yellow.

Our theoretical results indicate that decanoic acid forms water-filled stable vesicles whose properties depend on the environmental temperature. On the other hand, several theories indicate that primitive transmembrane peptides could develop functional properties on either side of the primitive compartments required for life to occur. Thus, we decided to look further into the interaction between DA vesicles and primitive-like peptides under different temperatures. The model peptide comprises 16 hydrophobic amino acid residues that fold as an alpha-helix secondary structure. The sequence selection was based upon their preponderance in the life origin era and induced the peptide diffusion into the DA bilayer. Twelve system peptide/DA-monomer-containing systems (DAP systems) were made and analyzed as described above (Figure 3). 

**Figure 4:**
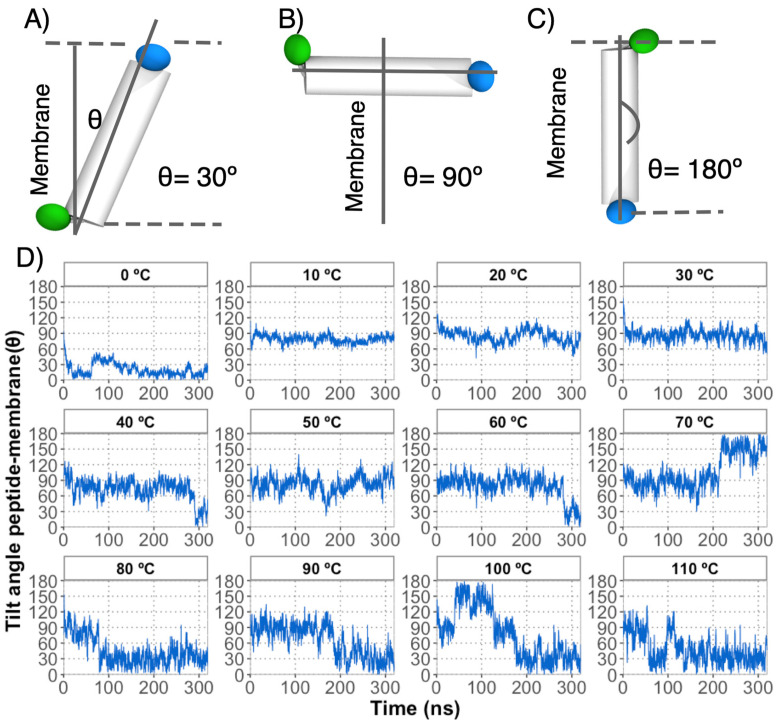
Definition of tilt angle between the peptide axis and the bilayer norm vector. (**A**) The peptide vector was defined by the bond angle between the alpha carbons of residues 1 (blue) and 16 (green). To avoid curvature perturbations, the decanoic acids located near 8 Å from the peptide were the reference to the bilayer norm vector. This approach allowed the evaluation of the angle between the peptide and bilayer, where angles close to 0° represent the insertion of the peptide from the C-terminus to N-terminus (**A**), angle values close to 90° exhibit a peptide over the bilayer surface (**B**), and angles close to 180° are related to the peptide insertion from the N-terminus to C-terminus. (**C**). The tilt angle of the peptide-membrane exhibits a dynamic range between 0° and 180° across the trajectories (**D**).

Although the trajectories show that the vesicles are formed in the temperature range tested, below 30 degrees they adopt a more defined spheroid shape, with a lower proportion of monomers adopting a liquid phase than equivalent DA-only simulations. Furthermore, the peptide affects both bilayer leaflets independently of the temperature assayed. Additionally, increased disorder and structural deformation near the peptide are observed. Interestingly, the peptide remains in the bilayer surface below 70 °C, and the internal DA layer appears to be less affected by the embedded peptide, while higher temperatures increase the peptide diffusion and the vesicle structure (Table S1, Figure S3 in Supplementary Materials). Finally, the time evolution of the tilt of the peptide axis with respect to the bilayer patch normal axis indicates that the bilayer asymmetry may result from the fact that the peptide does not adopt a similar or stable transmembrane orientation across different systems. It should be noted that below 10 degrees the peptide remains in its initial position (randomly defined) due to the phase adopted by the membrane, which correlates with the lower diffusion constant observed (Figure 4).

## 4. Conclusions

Understanding the self-assembly process of SCFAs and their capacity to generate water-filled compartments at prebiotic temperatures could provide valuable insights into how early earth conditions could generate life. We studied DA vesicles using atomic-level molecular dynamics simulations, from which structural and dynamic information was obtained. The simulation length of MD is directly limited by the number of atoms simulated; thus, we studied vesicles that were somewhat smaller than those obtained experimentally. With this theoretical approach, we observed the formation of small DA water-filled vesicles on a nanosecond time scale, the structure and dynamical properties of which were clearly affected by the temperature. At lower temperature regimes (0 °C to 20 °C), the DA bilayer adopts a clearly gel-like phase, transitioning from a ripple to a more defined liquid phase above 30 °C. According to the trajectory analysis, DA bilayers have spherical morphologies, and their compartment and membrane permeability varies with temperature. Furthermore, prebiotic-like peptides could modify the bilayer phase or disrupt the bilayer permeability when adopting a transmembrane orientation.

Our results raise questions and suggest experiments that are currently underway, such as the atomic-level effect of higher salt conditions, the protonation states, or mixed SCFAs. Finally, the theoretical approach presented here could help in understanding the incorporation of other prebiotic molecules into primitive amphiphile membranes [1,37] or their further application beyond the astrobiology field.

## Figures and Tables

**Table 1:** Simulated DA self-assemblies at different temperatures.

Sample *	Temperature °C	Radius (nm)	Thickness (nm)	% Inner Leaflets DA Monomers	3D Diffusion Constant **	SASA (nm^2^)
DA-0	0	3.55	1.44	20.9	0.89	5230
DA-10	10	3.51	1.51	22.3	0.98	5219
DA-20	20	3.53	1.51	21.4	1.77	5523
DA-30	30	3.56	1.55	22.5	1.75	5784
DA-40	40	3.61	1.52	22.4	1.39	6173
DA-50	50	3.51	1.42	25.6	1.42	5883
DA-60	60	3.67	1.54	21.9		6802
DA-70	70	3.59	1.45	23.4		6747
DA-80	80	3.61	1.47	24.2		7051
DA-90	90	3.59	1.45	23.4		7119
DA-100	100	3.61	1.47	23.0		7473
DA-110	110	3.64	1.51	21.4		7942

* Molecular dynamics trajectory. ** At temperatures exceeding 50 °C, the trajectories of the molecular systems exhibit an anomalous diffusion regime, and the corresponding diffusion coefficients are not exhibited.

## Data Availability

Data are available on demand to the authors.

## Supplementary Materials

The following supporting information can be downloaded at: https://www.mdpi.com/article/10.3390/membranes13050469/s1. Figure S1: Order parameter of decanoic acid acyl chain; Figure S2: Density maps of decanoic acid headgroups (Oxygens from carboxylic heads) along DA trajectories; Figure S3: Effect of temperature on radial distribution function profiles from different pairs of atoms of dec-anoic acids and the peptide studied in trajectories; Table S1: Simulated DA self-assemblies with peptide at different temperatures.
