# Supplementary material for: Insights into Early Steps of Decanoic Acid Self-Assemblies under Prebiotic Temperatures Using Molecular Dynamics Simulations
Source: Membranes (Basel). 2023 Apr 28;13(5):469. doi: 10.3390/membranes13050469 (PMC10223666; doi:10.3390/membranes13050469)
Supplement: Supplementary file 1 [file membranes-13-00469-s001.zip › Reviewed_membranes-2306622-supplementary.pdf]

membranes

Supplementary Materials

Insights into Early Steps of Decanoic Acid Self-Assemblies under Prebiotic Temperatures Using Molecular Dynamics Simulations

Romina V. Sepulveda [1,*], Christopher Sbarbaro [1], Ma Cecilia Opazo [2], Yorley Duarte [1], Fernando González-Nilo [1] and Daniel Aguayo [1,3*]

[1] Center for Bioinformatics and Integrative Biology, Facultad de Ciencias de la Vida, Universidad Andres Bello, Av. República 330, Santiago 8370146, Chile
[2] Instituto de Ciencias Naturales, Facultad de Medicina Veterinaria y Agronomía, Universidad de Las Américas, Manuel Montt 948, Providencia 7500000, Chile.
[3] Agricultura Digital, Servicio Agrícola, Salinas y Fabres S.A., Ruta 5 Sur, Parcela 165, Hijuela Larga, Paine 9540000, Chile
* Correspondence: romina.sepulveda@unab.cl, daniel.aguayo.cl@gmail.com

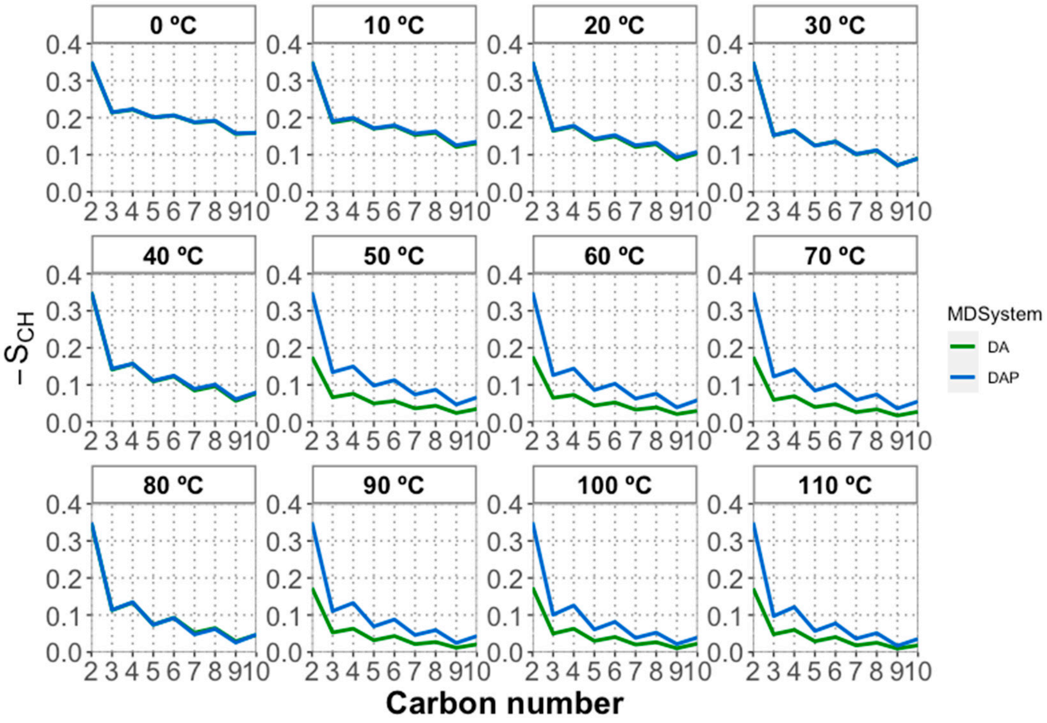

Figure S1. Order parameter of decanoic acid acyl chain. Decanoic Acid systems (green) and Decanoic Acid/peptide (blue). The temperature promotes a fallen in the values as a consequence of the motion of acyl chains.

Table S1. Simulated DA self-assemblies with peptide at different temperatures.

Sample*	Temperature °C	Radius (nm)	Thickness (nm)	% inner Leaflets DA monomers	3D Diffusion constant	SASA (nm²)
DAP-0	0	3.50	1.47	21	1.28	4959
DAP-10	10	3.49	1.46	24.3	2.29	5051
DAP-20	20	3.53	1.55	20.2	3.73	5395
DAP-30	30	3.57	1.52	22.9	6.39	5804
DAP-40	40	3.58	1.56	21.8	8.06	5992
DAP-50	50	3.56	1.47	22.7	1.48	6174
DAP-60	60	3.54	1.42	25.9		6309
DAP-70	70	3.69	1.55	21.4		7102
DAP-80	80	3.59	1.45	24.9		6902
DAP-90	90	3.62	1.47	23.1		7313
DAP-100	100	3.61	1.47	22.5		7524
DAP-110	110	3.64	1.51	21.5		7913

* Molecular dynamics trajectory.

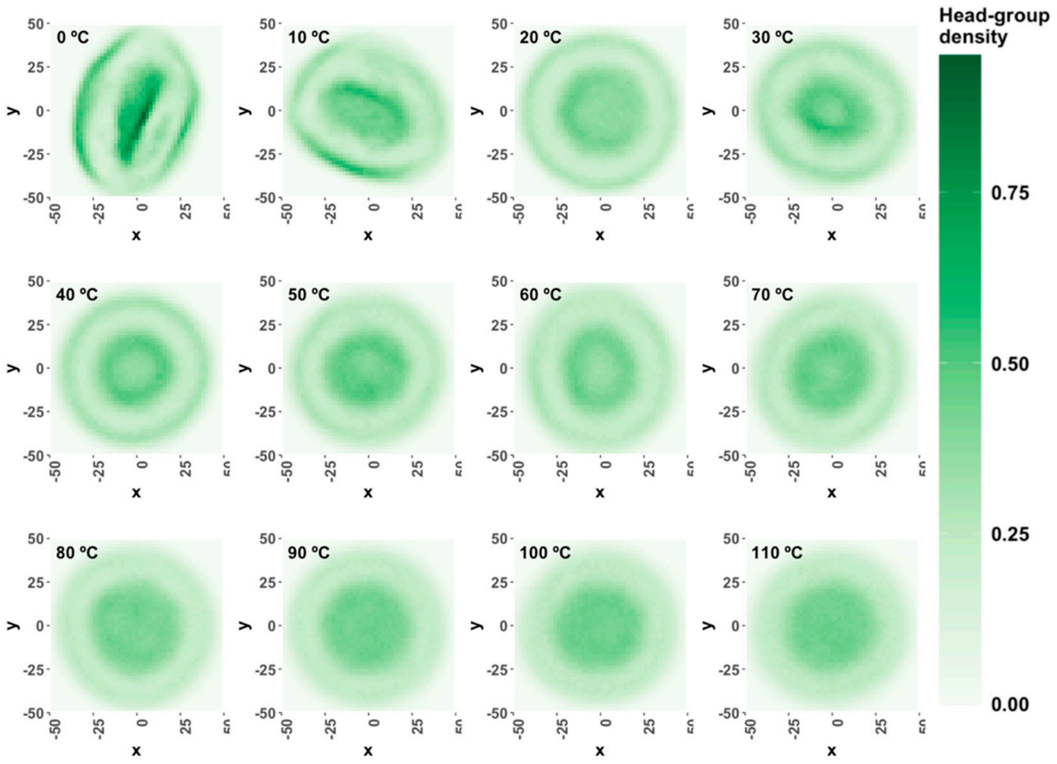

Figure S2. Density maps of decanoic acid headgroups (Oxygens from carboxylic heads) along DA trajectories. Each plot represents the measurement at different temperatures. The density value was calculated by x and y axis, and the color intensity represents the frequency of the headgroups along trajectories.

1.

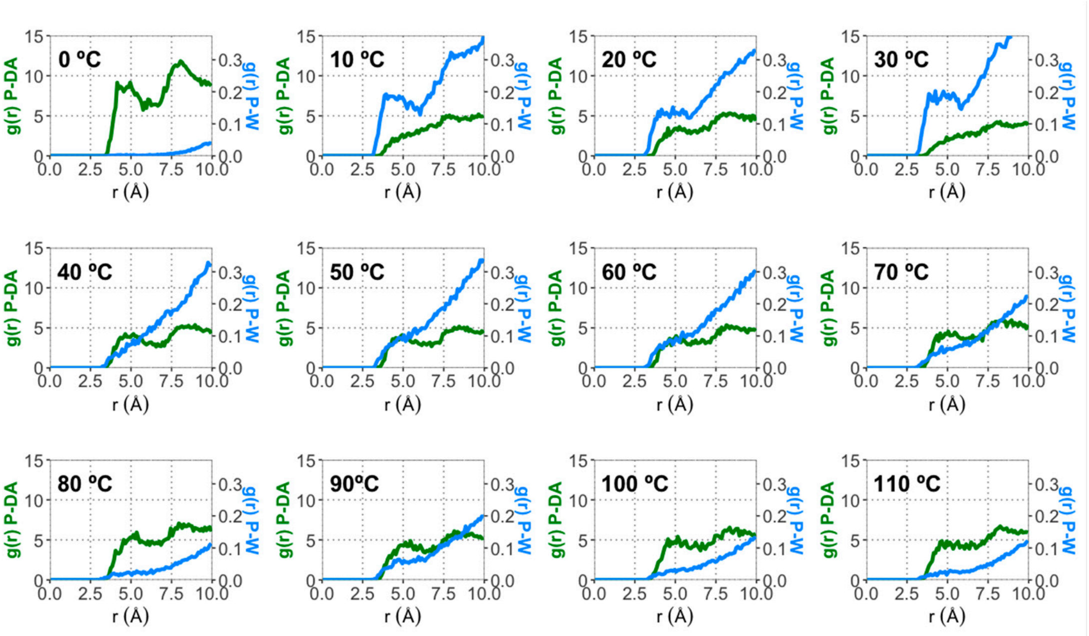

Figure S3. Effect of temperature on radial distribution function profiles from different pairs of atoms of decanoic acid and the peptide studied in trajectories. The green line represents g(r) from C-alpha from residues 7 and 8 of peptide and carbon 10 of the decanoic acid head (P-DA). The blue line represents the g(r) from C-alpha from residues 7 and 8 of peptide and waters (P-W). Combining the two RDF patterns could lead to an understanding peptide penetration into the bilayer membrane.
